# Comparative study of ionic currents and exocytosis in hair cells of the basilar and amphibian papilla in bullfrogs

**DOI:** 10.3389/fncel.2022.1064886

**Published:** 2023-01-09

**Authors:** Jingjing Zhao, Ning Yin, Geng-Lin Li

**Affiliations:** ^1^ENT Institute and Department of Otorhinolaryngology, Eye and ENT Hospital, Fudan University, Shanghai, China; ^2^NHC Key Laboratory of Hearing Medicine, Fudan University, Shanghai, China; ^3^State Key Laboratory of Medical Neurobiology and MOE Frontiers Center for Brain Science, Fudan University, Shanghai, China

**Keywords:** amphibian papilla, basilar papilla, hair cell, Ca^2+^ and K^+^ current, exocytosis, synaptic vesicle, readily releasable pool

## Abstract

Hearing organs in the peripheral of different vertebrate species are extremely diverse in shape and function. In particular, while the basilar papilla (BP) is elongated and covers the sounds of both low and high frequencies in turtles and birds, it is round and responds to high frequencies only in frogs, leaving the low frequencies to the amphibian papilla (AP). In this study, we performed patch-clamp recordings in hair cells of both hearing organs in bullfrogs and conducted a comparative study of their ionic currents and exocytosis. Compared to hair cells in AP with a large tetraethylammonium (TEA)-sensitive slow-activating K^+^ current (*I*_K_), those in BP exhibited a small 4-aminopyridine (4-AP)-sensitive fast-inactivating K^+^ current (*I*_A_). Furthermore, hair cells in BP exhibited a significantly smaller Ca^2+^ current with a more positive half-activation voltage (V_half_) and a slower slope of voltage dependency (*k*). In response to step depolarization, exocytosis (ΔC_m_) in BP hair cells was also significantly smaller, but the Ca^2+^ efficiency, assessed with the ratio between ΔC_m_ and Ca^2+^ charge (Q_Ca_), was comparable to that of AP hair cells. Finally, we applied a paired-step depolarization and varied the interval in between, and we found that the replenishment of synaptic vesicles was significantly slower in BP hair cells. Together, our findings suggest that hair cells tuned to high frequencies in bullfrogs release less synaptic vesicles and recycle synaptic vesicles more slowly, allowing them to cope well with the large DC component found in their receptor potentials *in vivo*.

## Introduction

A hallmark characteristic of the auditory systems in different vertebrate species is their tonotopic organization of hair cells and afferent fibers. Auditory organs are intrinsically determined by a position-dependent variation in the biophysical properties of the hair cells (Fettiplace and Fuchs, 1999), which are perfectly propitious to fine intensity discrimination over a wide dynamic range. Various sound frequencies are distributed along the cochlea axis, and each hair cell is tuned to a narrow frequency range that is known as the characteristic frequency, the sound frequency at which a cell responds maximally. The bullfrog’s inner ear contains three sensory organs for auditory reception: the saccule (S), amphibian papilla (AP), and basilar papilla (BP). Of these, AP and BP have been proven to be particularly sensitive to airborne sound. AP has a diamond-shaped rostral end and a long, narrow caudal extension (Lewis, 1984), with a broad frequency response ranging from 100 to ∼1,250 Hz (Lewis, 1981; Lewis et al., 1982). AP has two identified distinctive populations of hair cells, an oscillatory-type electrically tuned hair cell that dominates the low- to mid-frequency auditory range of the AP, and a non-oscillatory cell type that dominates the mid- to high-frequency region (Smotherman and Narins, 1999a), with the lowest frequencies being encoded by rostral hair cells and progressively higher frequencies being transduced more caudally (Lewis et al., 1982). Although AP lacks the flexible basilar membrane, it possesses a tapered tectorial membrane that might play a similar role (Lewis and Leverenz, 1983), displaying comparable frequency selectivity. While BP is a tubular evagination of the saccule, terminating in a thin contact membrane separates endolymphatic and perilymphatic spaces (Frishkopf and Flock, 1974). The sensory epithelium of BP is approximately 100 μm long and includes 50–100 hair cells (Smotherman and Narins, 1999b), which are the smallest ones in the frog auditory system, with cell bodies normally less than 20 μm in diameter. BP is responsible for encoding the upper limits of the bullfrog’s spectral sensitivity (Feng et al., 1975), responding to the sounds at frequencies above 2 kHz.

Hair cells of all vertebrate species have specialized synaptic ribbons that facilitate high rates of sustained synaptic transmission and coordinated release of plentiful vesicles in the vicinity of the active zone (Glowatzki and Fuchs, 2002; Sterling and Matthews, 2005; Keen and Hudspeth, 2006; Goutman and Glowatzki, 2007). When Ca^2+^ enters through the voltage-gated channels, it triggers the vesicle fusion to the basolateral membrane, and glutamate is released onto afferent terminals. Indeed, hair cell afferent synapses rely on a rapid vesicle pool refilling to continuously encode sound information. The great synaptic responses require the pool of docked vesicles closed to the plasma membrane, which are immediately available for exocytosis and termed a readily releasable pool (RRP), turning over entirely at least five times over the course of stimulus (Patel, 2013). Synaptic ribbons rapidly recruit the vesicles from one or more larger pools that are located far away from the Ca^2+^ channels (Von Gersdorff et al., 1996; Moser and Beutner, 2000; Schnee et al., 2005), playing the role of RRP replenishment.

Hair cells of all vertebrate species possess a variety of ionic channels in their plasma membrane, which are involved in many functions, including mechanoelectrical transduction, electrical frequency tuning, and synaptic transmission. According to the earlier findings on bullfrog (Hudspeth, 1986), the lateral membrane of vestibular hair cells contains a calcium-dependent potassium channel (K_Ca_), A-type potassium channel (K_A_), delayed rectifier potassium channel (K_DR_), inward rectifier potassium channel (K_IR_), L-type calcium channel, and other channels. The outward current is predominantly *I*_K(Ca)_ in rostral and caudal hair cells of AP (Smotherman and Narins, 1999a), which is rapidly activating and calcium-dependent. The majority of hair cells also have a slowly activating, outwardly delayed rectifying, and voltage-dependent potassium current (*I*_K_) (Smotherman and Narins, 1999b). An identified fast-inactivating within tens to hundreds of milliseconds and voltage-gated outward current *I*_A_, which replaces *I*_K_ in a small subset cell (Smotherman and Narins, 1999b), has been described in hair cells of the frog sacculus (Hudspeth and Lewis, 1988) and in the low- to mid-frequency region of the leopard frog AP (Smotherman and Narins, 1999a). Only rostral hair cells exhibited an inactivating potassium current *I*_A_, whereas an inwardly rectifying potassium current (*I*_K(IR)_) was confirmed only in caudal AP hair cells (Smotherman and Narins, 1999a).

Previously, ionic currents in BP hair cells have been studied only in dissociated cells, and examination of exocytosis from BP hair cells is completely lacking. To address this, we decided to study the ionic currents and exocytosis in both AP and BP hair cells, side by side. We first used K^+^ channel blockers and characterized the different types of potassium current in hair cells and then compared directly the features of their Ca^2+^ currents. Second, we monitored the ΔC_m_ of hair cells during stimulation using a whole-cell patch-clamp, so as to investigate the tonotopic differences in synaptic vesicle exocytosis, including the kinetics of vesicle pool release and refilling. Finally, we applied a paired-pulse stimulation to calculate C_m_ recovery ratio and evaluate the synaptic vesicle replenishment function. Our results show that *I*_K(Ca)_ plays a predominant role in the outward current of both medial AP and BP hair cells. Concurrently, we identified *I*_K_ accounting for a large proportion of the AP hair cells, which is sensitive to tetraethylammonium (TEA). While in BP hair cells, *I*_A_ is a characteristic type of potassium current and is of high sensitivity to 4-AP. Additionally, hair cells from medial AP release more synaptic vesicles for both short and long step stimulations with more Ca^2+^ influx, along with a notably larger RRP and SRR, and a faster replenishment of synaptic vesicles.

## Materials and methods

### Electrophysiology

**FIGURE 1:**
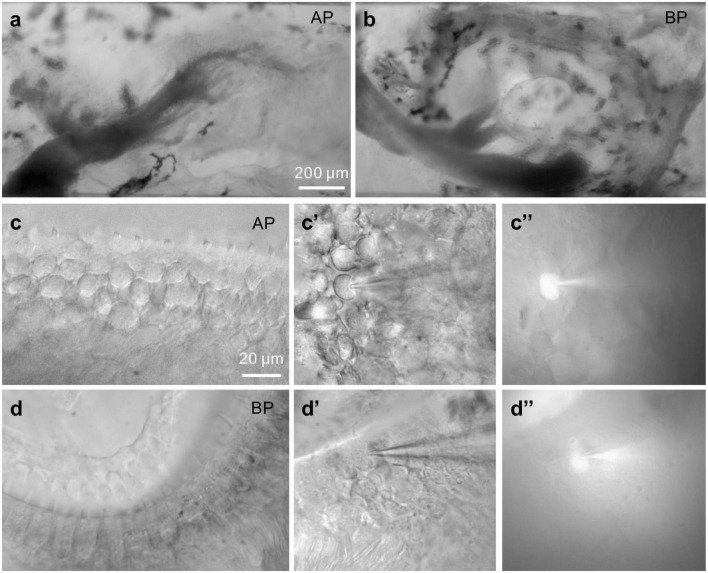
Images of bullfrog’s AP and BP. **(a,b)** Shown above are the amphibian papilla (AP) **(a)** and basilar papilla (BP) **(b)** preparation under a 10× microscope, respectively. **(a)**: In this view of AP, rostral is to the right, caudal to the left. The sensory epithelium is visible and runs horizontally. Recordings were made from hair cells in the mid-regions of the AP, which responds to the sounds at frequencies of approximately 250–750 Hz. The scale bar is approximately 200 μm. **(c,d)** Displayed are the higher magnification images of medial AP **(c)** and BP **(d)** hair cells under a 60× microscope. **(c’,d’)** Bright-field images of hair cells from the medial AP **(c’)** and BP **(d’)** under voltage-clamp. A recording pipette filled with Alexa488-internal solution is on the right, approaching and patching the hair cell. Then, hold on for several minutes. The fluorescent cells **(c,”d”)** are indicative of internal solution influx, successfully patching the given cells (bars: 20 μm).

Adult American bullfrogs (*Rana catesbeiana*) were obtained from a local vendor. Bullfrogs in the weight range of 200–300 g were used for the study. Before being pithed and decapitated, the bullfrogs had been anesthetized in an ice bath for 20 min. Then, the tissues of AP and BP were carefully dissected out (refer to Figure 1) in an oxygenated extracellular solution containing (in mM): 95 NaCl, 2 KCl, 2 CaCl_2_, 1 MgCl_2_, 3 D-glucose, 1 creatine, 1 Na-pyruvate, and 10 HEPES. For outward K^+^ current recording, an additional 2 mM CdCl_2_ was included. The osmolality was adjusted to 230 mOsm with NaCl and pH 7.40 with NaOH. Then, they were transferred and settled into a recording chamber containing about 1 ml of extracellular solution for the following experiment. Chemicals and salts were purchased from Sigma.

The recording chamber, along with the tissue, was placed under an upright microscope (Olympus) equipped with a 60 × water-immersion objective. Whole-cell patch-clamp recordings were performed at room temperature (∼23°C) within 2 h of dissection, using an EPC10/2 patch-clamp amplifier (HEKA Electronics, Lambrecht Pfalz, Germany) and Patchmaster (HEKA Electronics) was used to generate a voltage- or current-clamp commands to drive the amplifier. The hair cells were held at −90 mV. The liquid junction potential of −10 mV was corrected offline and data were corrected by subtracting 10 mV from all potentials.

Recording micropipettes were made from borosilicate glass tubes (BF150-86-10, Sutter Instruments, Novato, CA, USA) using a two-stage vertical pipette puller (Narishige, Tokyo, Japan) to tip diameters of ∼1 μm. The electrodes were coated with dental wax to reduce stray capacitance and enhance C-fast compensation. A Cs + -based internal solution was used to block K^+^ current and isolate Ca^2+^ current. Pipettes were filled with an internal solution containing (in mM): 80 Cs-gluconate, 20 CsCl, 10 TEA-Cl, 2 EGTA, 3 Mg-ATP, 0.5 Na-GTP, and 10 HEPES (230 mOsm, pH 7.30). The internal solution for outward K^+^ current recording contained the following: 80 K D-gluconate, 30 KCl, 2 EGTA, 3 Mg-ATP, 0.5 Na-GTP, and 10 HEPES. Pipettes were pulled to resistances of 4 to 7 MΩ. Series resistances (Rs) during recordings typically ranged from 10 to 30 MOhm, continuously compensated using the amplifier’s compensation circuitry. For K^+^ current recording, voltage steps were delivered from the holding potential of −90 mV to potentials ranging from −125 to + 45 mV in 10 mV increments, in order to obtain the current-voltage relationship (I-V curve). To verify the pharmacological identity of different potassium currents, we applied the more positive holding potential (−65 mV), with the depolarization voltage steps ranging from −125 to + 5 mV in 10 mV increments.

For Ca^2+^ current recording, a voltage ramp of 300 ms from −90 to + 70 mV was applied to hair cells to evoke Ca^2+^ currents, and the peak of this Ca^2+^ current (I_Ca_) was determined. We then fitted the current-voltage relationship (I-V curve) with a Boltzmann equation as below to obtain the half activation potential (V_half_) and the slope factor (*k*), which reflects the steepness of voltage dependence in Ca^2+^ current activation. Where V is the command membrane potential, V_rev_ is the reversal potential and G_max_ is the maximum chord conductance.


$$\text{I}\,\left(\text{V}\right)=\left(\text{V}-{\text{V}}_{\text{rev}}\right)\times \frac{{\text{G}}_{\text{max}}}{1+\text{exp}\,\left(-\left(\text{V}-{\text{V}}_{\text{half}}\right)/k\right)}$$

For membrane capacitance (C_m_) measurement, the “Sine + DC” technique (Lindau and Neher, 1988) in Patchmaster was applied under a voltage clamp, using a software lock-in amplifier (Lindau and Neher, 1988; Gillis, 2000). Once forming a tight seal, each hair cell was held at −90 mV and driven with sine waves of 1 kHz (25 mV in amplitude) to determine the C_m_ (Li et al., 2009). The real-time changes in averaged C_m_ [ΔC_m_ = C_m (response)_–C_m (baseline)_], evoked by cell membrane depolarization, were used to generally assess and quantify the total synaptic vesicle exocytosis from hair cells.

For the exocytosis dynamic assay, ΔC_m_ was measured at variant stimulation lengths from 2, 5, 10, 20, 50, 100, 200, 500 to 1,000 ms and plotted against stimulation time (t) recorded from individual hair cell, and the data points were fitted to a combination formula, consisting of a single exponential function for the release of the readily releasable pool (RRP) of synaptic vesicles (C_m,RRP_, τ_RRP_) and a linear function for the sustained release of synaptic vesicles (R_sustained_, SRR):


$$\Delta \,C_{\text{m}}\,\,\left(\text{t}\right)={\text{C}}_{\text{m},\text{RRP}}\,\cdot \,\left(1\,-\exp\,\left(\frac{\text{t}}{\tau_{\text{RRP}}}\right)\right)\,+R_{\text{sustained}}\,\cdot t$$

Then, the numbers of synaptic vesicles were estimated with the capacitance values, using a conversion factor of 37 aF/vesicle (Lenzi et al., 1999; Johnson et al., 2009).

For synaptic vesicle replenishment assessment, a paired-pulse of 100 ms was executed with various interpulse intervals (100, 200, 500, 1,000, and 2,000 ms). The average ratio of ΔC_m_ (ΔC_*m*2_/ΔC_m1_) was calculated to evaluate the C_m_ recovery.

### Statistical analysis

Data analysis was performed using Igor Pro (WaveMetrics, USA) and Prism Software (GraphPad, USA) with built-in macros and functions. Statistical comparisons of means were assessed with the two-tailed paired or unpaired Student’s *t*-test for single measurements. For multiple measurements, two-way analysis of variance (ANOVA), followed by the Bonferroni *post-hoc* test for multiple comparisons, was used to compare the data sets between AP and BP groups. Data were expressed as mean ± SEM with *p*-values of < 0.05 which is considered statistically significant. For the main results, Cohen’s *d* was used to accompany the reporting of *t*-test and ANOVA results, which is an appropriate effect size for the comparison between two means. Cohen’s *d* can be calculated as the difference between the means divided by the pooled SD, and some minimal guidelines are that *d* = 0.2 indicates a small effect, *d* = 0.50 indicates a medium effect, and *d* = 0.80 indicates a large effect.

## Results

The optimal stimulus frequency varied in a systematic way along the sensory epithelium; hair cells in the cochlea form a tonotopic axis, for instance. In the AP organ, rostral hair cells responded best to stimulation at frequencies as low as 150 Hz, while caudal cells showed peak responses up to 700 Hz (Patel et al., 2012). In this study, we chose the hair cells located in the medial region of AP epithelium, rather than from the caudal or rostral portion, to record the electrical properties, approximately corresponding to the frequency range of 250–750 Hz (Smotherman and Narins, 1999a). To cover possible subtypes of cells at different locations, we randomly chose the cells in the epithelium of BP for patch-clamp recording.

Adult hair cells from AP and BP epithelium shown in Figures 1a–d are classified as the lower and higher frequency ones, respectively, which were selected to explore the tonotopic variation in synaptic exocytosis. The hair cells are loaded with a fluorescent dye through the recording electrode, confirming that the internal solution has entered into the given cell (Figures 1c,”d”).

### Potassium currents in BP and AP hair cells

**FIGURE 2:**
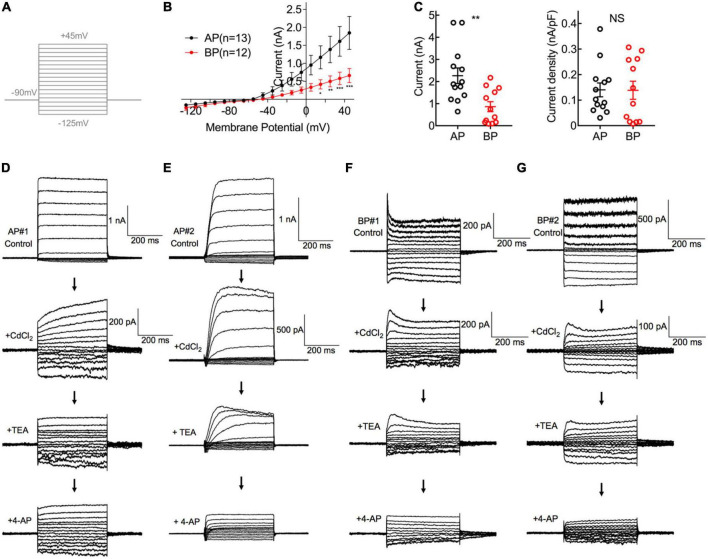
Comparisons of K^+^ Current in AP and BP hair cells. **(A)** The voltage-clamp protocol used in potassium current recordings. Activation of K^+^ currents was elicited by a series of 100-ms depolarizing voltage steps (command potentials between –125 and + 45 mV, in 10 mV increments) from a holding potential of –90 mV. **(B)** Corresponding I–V curves are plotted for whole-cell potassium currents at the different test potentials from medial AP (black) and BP (red) hair cells. AP hair cells showed a notably larger amplitude of total outward current than that of BP. **(C)** The left panel showed the peak potassium current at the potential of + 45 mV in medial AP and BP hair cells (*n* = 13 and 12; unpaired Student’s *t*-test, **p < 0.01). The right panel showed no significant difference in the density of potassium current. **(D–G)** A different protocol with voltage steps ranging from –125 to + 5 mV, from a holding potential of –65 mV, was used in pharmacological experiments to dissect different types of potassium currents. Displayed are the diversity of representative potassium currents recorded from two medial AP **(D,E)** hair cells and two BP **(F,G)** hair cells, by the successive application of three blockers, 2 mM CdCl_2_, 10 mM tetraethylammonium (TEA), and 1 mM 4-AP. **(D)** Here shows a representative cell of the majority (20/23) tested medial AP hair cells, which exhibited a slowly non-inactivating, delayed rectifying outward current (top panel) with increasing hyperpolarization, accompanied by a non-inactivating inward rectifying potassium current (*I*_K(IR)_) that has been identified in saccular hair cells. Note that 10 mM TEA can reduce the component of non-inactivating outward current. **(E)** Displayed is one of the minority (3/23) hair cells recorded from medial AP hair cells, which shows a fast inactivating component in outward current and it can be blocked by applying 4-AP. **(F,G)** Typical traces of potassium current from BP hair cells, which depicted low sensitivity to TEA and high sensitivity to 4-AP.

Membrane depolarization from −125 to +45 mV is capable of activating a combination of *I*_Ca_, *I*_K(Ca)_, *I*_K_, *I*_A_, and *I*_K(IR)_ (Hudspeth and Lewis, 1988) in standard saline (Figure 2A). In our study, the external addition of 2 mM Cd^2+^ can completely block the inward *I*_Ca_ and therefore largely and rapidly eliminate the calcium-dependent outward potassium current *I*_K(Ca)_ (Smotherman and Narins, 1999a). TEA can block the voltage-dependent slowly activating potassium current *I*_K_, and 4-AP serves as an A-type and delayed rectifier-type channel blocker (Nenov et al., 1997), has been reported to specifically eliminate *I*_A_ in hair cells (Lewis and Hudspeth, 1983), and probably affects K^+^ channels other than *I*_K(Ca)_ (Ricci et al., 2000).

First of all, we recorded the control potassium current without any blockers and noticed that the amplitude of potassium in medial AP hair cells was significantly larger than that in BP [*F*(1,22) = 5.16, *p* = 0.03, two-way ANOVA followed by Bonferroni *post-hoc* test; Figure 2B]. Besides, the steady-state (averaged over the last 100 ms of pulse) current amplitudes of a depolarizing step to + 45 mV differed significantly between AP and BP hair cells, with the means of 2.264 ± 0.350 pA and 0.864 ± 0.227 (*p* = 0.003, Cohen’s *d* = 1.331; Figure 2C, left panel), respectively. Considering the variance in cell size, we measured the capacitance membrane of hair cells from both AP and BP and found that AP hair cells have a significantly larger C-slow value than BP hair cells (17.2 ± 1.59 pF vs. 7.55 ± 0.995 pF, *n* = 13 and 12 for the two groups; unpaired Student’s *t*-test, *p* < 0.001). Then, we calculated the density of potassium current and found no significant difference in the two cell types (AP: 0.140 ± 0.027 nA/pF, BP: 0.139 ± 0.035 nA/pF, *p* = 0.97, Cohen’s *d* = 0.015; Figure 2C, right panel).

To further identify the different components of potassium current, we then applied various blockers. Given *I*_K(Ca)_ was found to be sensitive to the external calcium concentration, we first added CdCl_2_ to the recording solution and observed that 2 mM CdCl_2_ routinely reduced the amplitude of the net outward current in AP, with the amplitude decreased averagely from 1.66 ± 0.416 nA to 0.192 ± 0.041 nA (*p* = 0.02, paired *t*-test; Cohen’s *d* = 3.130), approximately 88.43% of reduction. The sensitivity to external cadmium suggests that the slowly non-inactivating outward current is a calcium-dependent potassium current, *I*_K(Ca)_, and it accounts for the majority of the outward current in medial AP hair cells. In BP hair cells, the external CdCl_2_ reduced the average amplitude from 0.736 ± 0.191 nA to 0.145 ± 0.042 nA (*p* = 0.03, paired *t*-test; Cohen’s *d* = 4.469), approximately 80.32% of reduction of amplitude, which is comparable to medial AP hair cells.

In the next place, owing to the outward current appearing in the presence of cadmium which is *I*_K_, we subsequently applied TEA to verify its pharmacological effect on it. We found that 10 mM TEA significantly reduced the non-inactivating component in AP hair cells, with the steady-state amplitude decreased from 0.215 ± 0.056 nA to 0.147 ± 0.039 nA (*p* = 0.04, paired *t*-test; Cohen’s *d* = 0.705) on average, with approximately 31.63% of reduction. In contrast, TEA had no effect on the amplitude of the non-inactivating outward current in BP hair cells, with the average amplitudes before and after adding TEA, which was 0.31 ± 0.106 nA and 0.204 ± 0.053 nA, respectively (*p* = 0.27, paired *t*-test).

Finally, the effect of 1 mM 4-AP was assessed on the fast inactivating component of the outward current (*I*_A_). We noticed that of all medial AP hair cells in the study (*n* = 23), the majority (*n* = 20) showed the non-inactivating potassium currents (Figure 2D), and only three cells showed the typical inactivating potassium currents (Figure 2E). Whereas in all tested BP hair cells (*n* = 13), there was a rapidly inactivating outward current (Figures 2F, G), which could be blocked by 4-AP, confirming the universal presence of A-type potassium current (*I*_A_).

Consistent with the previous findings (Smotherman and Narins, 1999a,b), our results revealed that in medial hair cells of AP, the outward current is predominantly *I*_K(Ca)_, which is rapidly activating and calcium-dependent and the majority also have a slowly activating, outwardly delayed rectifying and voltage-dependent potassium current (*I*_K_). Additionally, the earlier reports on frog crista ampulla that different types of potassium channels exist in the hair cells located in different regions and cells from the central region showed little or no *I*_A_ (Masetto et al., 1994; Russo et al., 1995), which is similar to our results about medial AP hair cells. While in the majority of BP hair cells tested, a rapidly activating and inactivating potassium A type current (*I*_A_) was identified and *I*_K(Ca)_ is similarly the dominant component in the outward potassium current.

### Calcium currents in BP and AP hair cells

**FIGURE 3:**
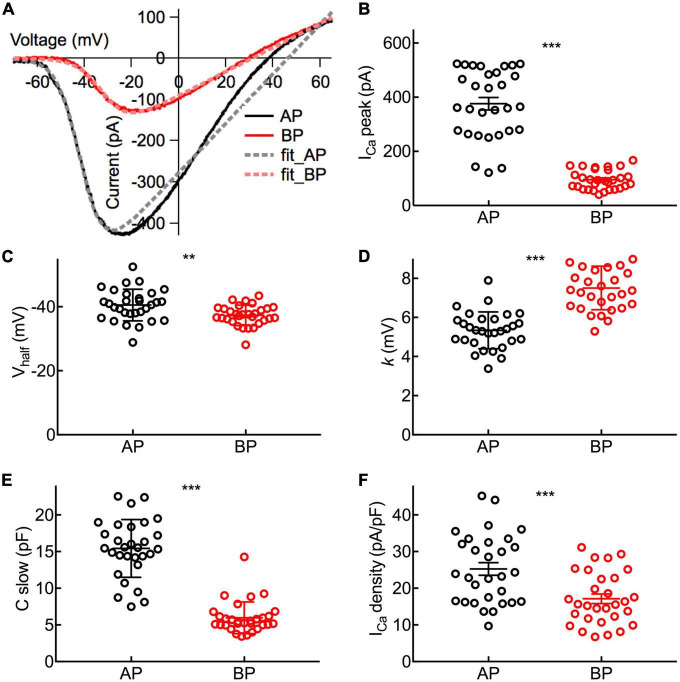
Properties of *I*_Ca_ in Hair Cells. **(A)** Representative I–V curves of the Ca^2+^ current (*I*_Ca_) recorded from an AP and a BP hair cell in response to a voltage ramp from -90 to + 70 mV under voltage-clamp and then leak subtracted. Continuous lines are fits obtained using a Boltzmann equation (in dashed lines). **(B)** Amplitude of *I*_Ca_ from near the peak of individual I–V curves (*I*_Ca_ peak). **(C)** Half activation potential (V_half_). **(D)** Slope of Ca^2+^ activation (*k*). **(B–D)**: The Ca^2+^ current in AP hair cells has a larger peak amplitude (*I*_Ca_ peak), a more negative half-activation voltage (V_half_), and a steeper voltage dependency (*k*). **(E)** The analysis of C-slow, which is deemed as the baseline membrane capacitance of hair cells. **(F)** Ca^2+^ current density calculated by *I*_Ca_ peak/C-slow. Data are presented as the mean ± SEM. ** means *p* < 0.01 and *** means *p* < 0.001, unpaired Student’s *t*-test; *n* = 30 hair cells for both AP and BP groups.

Depolarization of hair cells causes a Ca^2+^ entry, triggering transient C_m_ increments and elevations of global Ca^2+^ influx, which is controlled by the activation of the voltage-dependent Ca^2+^ current. We applied ramp stimulation and conducted whole-cell patch-clamp recordings (Figure 3A). Peak *I*_Ca_ is the maximal amplitude with all the calcium channels open, and it was significantly larger in medial AP hair cells (376 ± 23 pA, *n* = 30) than that of BP (95.4 ± 5 pA, *n* = 30; *p* < 0.001, unpaired Student’s *t*-test; Cohen’s *d* = 1.664, Figure 3B). Similarly, AP hair cells are almost three times larger than BP in size (C-slow value: 15.4 ± 0.718 pF vs. 5.97 ± 0.394 pF, *n* = 30; unpaired Student’s *t*-test, *p* < 0.001; Cohen’s *d* = 1.654, Figure 3E). We whereafter individually divided a peak *I*_Ca_ by the C-slow value to estimate the density of the current, which is correlated with the Ca^2+^ channel density in the single hair cell membrane. Not surprisingly, we found that the average density of *I*_Ca_ in each BP hair cell (17.1 ± 1.3 pA/pF, *n* = 30) was notably lower than that of AP (25.3 ± 1.72 pA/pF, *n* = 30) (unpaired Student’s *t*-test, *p* < 0.001; Figure 3F).

To characterize the functional properties of hair cells from the two auditory organs more comprehensively, V_half_ and *k* were figured out to depict the steepness of voltage dependence in Ca^2+^ channel activation. Concretely speaking, V_half_ describes the membrane potential at which the conductance is half activated while the slope factor (*k*) reveals the voltage sensitivity of activation. Noting *I*_Ca_ in AP hair cells has a more negative V_half_ (−40.5 ± 0.901 vs. −37.3 ± 0.579 mV, *n* = 30; unpaired Student’s *t*-test, *p* = 0.004; Cohen’s *d* = 0.772, Figure 3C) and a steeper activation slope (5.34 ± 0.172 vs. 7.51 ± 0.204 mV, *n* = 30; unpaired Student’s *t*-test, *p* < 0.001; Cohen’s *d* = 1.450, Figure 3D), suggesting that Ca^2+^ influx in AP hair cells is more sensitive to depolarization.

### Exocytosis in BP and AP hair cells

**FIGURE 4:**
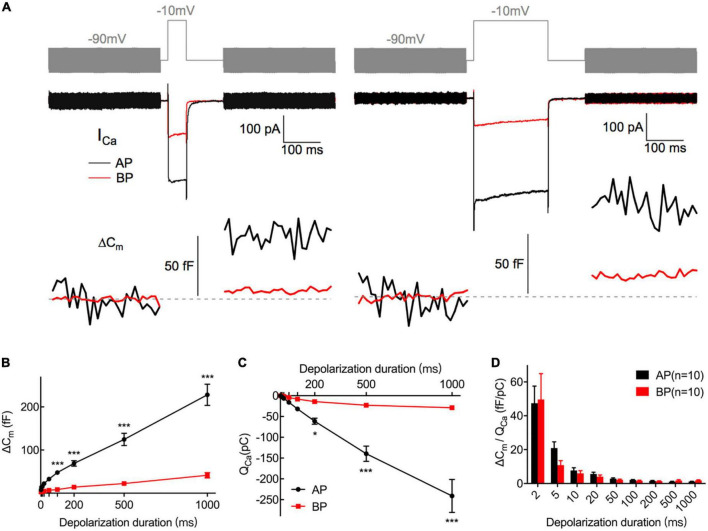
Exocytosis recorded by C_m_ measurements from hair cells. **(A)** Plotted are representative individual capacitance increase (ΔC_m_) and the corresponding *I*_Ca_ in an AP (black) and a BP (red) hair cell. The top trace (gray) shows the stimulus, in which the thick line represents a sine wave that is blanked during the depolarizing step. The middle trace shows *I*_Ca_; exocytotic ΔC_m_ traces are seen in the bottom panel, with the blank region representing a depolarizing step of 50 (left) and 200 ms (right), respectively. Horizontal dashed lines represent normalized C_m_ before stimulation. **(B,C)** ΔC_m_ values **(B)** and Ca^2+^ charge (Q_Ca_) **(C)** measured from AP and BP hair cells in response to increasing stimulation durations from 2 ms to 1 s, including 2, 5, 10, 20, 50, 100, 200, 500, and 1,000 ms. **(D)** Ca^2+^ efficiency in triggering exocytosis, assessed by the ratio of ΔC_m_/Q_Ca_, was found no significant difference in response to all the above stimulations. Values are presented as mean ± SEM and evaluated with two-way ANOVA followed by the Bonferroni *post-hoc* test. *p < 0.05, ***p < 0.001, ****p < 0.0001; *n* = 10 per group.

During exocytosis, synaptic vesicles fuse with the presynaptic membrane, increasing the whole-cell capacitance (Moser and Beutner, 2000). We, therefore, studied the exocytosis of AP and BP hair cells by monitoring the changes in their whole-cell capacitance (ΔC_m_) before and after voltage steps to elicit Ca^2+^ influx and exocytosis. A representative diagram of *I*_Ca_ and the corresponding ΔC_m_ in AP and BP hair cells of duration 50 ms (left) and 200 ms (right) is shown in Figure 4A, respectively. The induced ΔC_m_ was found to be significantly larger in AP than in BP hair cells [*F*(1,30) = 105, *p* < 0.001; Table 1 and Figure 4B]. Then, the pooled data for Ca^2+^ charge for all the stimulation durations were assessed, and we found that Q_Ca_ in BP hair cells was remarkably smaller [*F*(1,25) = 31.9, *p* < 0.001, two-way ANOVA followed by Bonferroni *post-hoc* test; Table 1 and Figure 4C], consistent with the findings shown in Figure 3B. The Ca^2+^ efficiency of exocytosis (qualitatively judged to be ΔC_m_/Q_Ca_) was found no significant difference for any pairwise comparison of means in response to all the stimulations from 2 to 1,000 ms (Table 1 and Figure 4D), indicative of a similar efficiency of Ca^2+^ influx in triggering exocytosis from AP and BP hair cells.

**TABLE 1:** Summary of ΔC_m_, Q_Ca_, and ΔC_m_/Q_Ca_ of AP and BP hair cells for various stimulation from 2 to 1,000 ms.

	Group	2 ms	5 ms	10 ms	20 ms	50 ms	100 ms	200 ms	500 ms	1,000 ms
ΔC_m_ (fF)	AP (*n* = 15)	7.75 ± 6.58	13.99 ± 8.77	15.32 ± 8.67	22.14 ± 10.18	32.74 ± 12.72	47.78 ± 17.49	68.92 ± 24.57	124.51 ± 54.70	228.13 ± 95.31
	BP (*n* = 17)	2.79 ± 3.13	3.57 ± 3.26	4.11 ± 2.08	4.88 ± 2.14	6.94 ± 3.46	8.33 ± 2.99	14.03 ± 10.78	22.32 ± 15.77	41.54 ± 24.78
	*P*-value	>0.99	>0.99	>0.99	0.70	0.08	<0.001	<0.001	<0.001	<0.001
Q_Ca_ (pC)	AP (*n* = 14)	−0.31 ± 0.16	−1.31 ± 0.55	−2.97 ± 1.11	−6.35 ± 2.54	−15.88 ± 6.89	−31.89 ± 13.26	−61.14 ± 27.33	−139.5 ± 68.87	−241.2 ± 148.96
	BP (*n* = 13)	−0.08 ± 0.05	−0.36 ± 0.16	−0.82 ± 0.34	−1.74 ± 0.73	−4.26 ± 1.79	−8.19 ± 3.23	−14.18 ± 6.33	−22.85 ± 12.58	−28.94 ± 19.96
	*P*-value	>0.99	>0.99	>0.99	>0.99	>0.99	>0.99	0.03	<0.001	<0.001
ΔC_m_/Q_Ca_	AP (*n* = 10)	47.42 ± 31.77	20.86 ± 11.59	7.67 ± 4.93	5.56 ± 3.32	2.83 ± 1.50	2.04 ± 0.82	1.49 ± 0.96	1.03 ± 0.68	1.10 ± 0.53
	BP (*n* = 10)	49.69 ± 48.13	10.86 ± 8.32	6.06 ± 4.65	4.07 ± 3.09	2.18 ± 1.35	1.58 ± 1.19	1.36 ± 0.76	1.76 ± 1.49	1.86 ± 1.03
	*P*-value	>0.99	>0.99	>0.99	>0.99	>0.99	>0.99	>0.99	>0.99	>0.99

Data are presented as the mean ± SD and n indicates the number of hair cells; *p*-values are presented in the table.

Ribbon synapses in hair cells can release the synaptic vesicles rapidly and continuously. When stimulation is turned on, hair cells release a small pool of synaptic vesicles rapidly, representing the exocytosis of the readily releasable pool (RRP) of synaptic vesicles docked at the active zones (Moser and Beutner, 2000; Beutner and Moser, 2001). Subsequently, hair cells are capable of releasing the synaptic vesicles continuously for as long as the stimulation is on, owning to the fast replenishment of synaptic vesicles from a refilling pool that is located further from the Ca^2+^ channels (Von Gersdorff et al., 1996; Von Gersdorff and Matthews, 1999).

**FIGURE 5:**
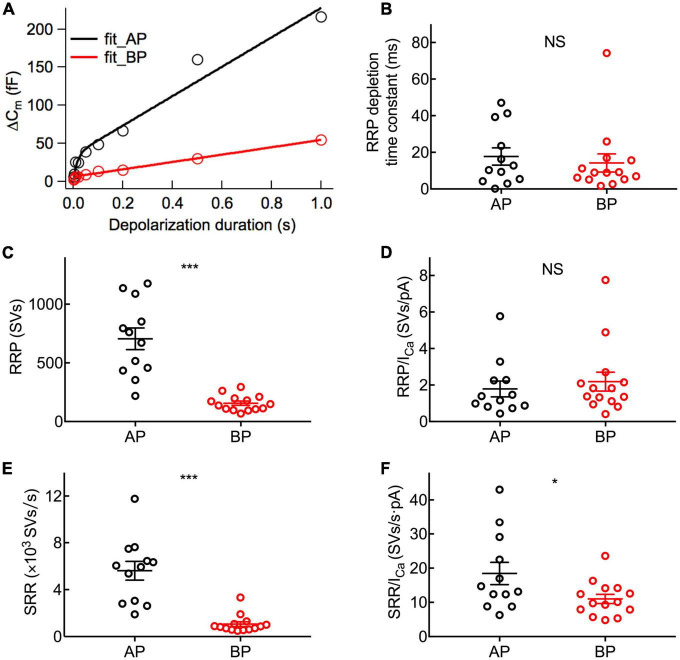
Dynamics of synaptic vesicle pools in hair cells. The kinetics of vesicle release was investigated by plotting and analyzing capacitance data against pulse duration from Figure 4. **(A)** Typical curves obtained by fitting the plots of ΔC_m_ against stimulus time for a pair of AP (black) and BP hair cells (red), respectively. Three parameters of exocytosis were extracted, including time constant (τ) to release RRP **(B)**, RRP **(C)**, and sustained release rate (SRR) **(E)** and in AP (*n* = 12) and BP (*n* = 14) hair cells. **(B)** There is no significant difference in the time constant to release RRP (17.7 ± 4.71 ms vs. 14.2 ± 4.92 ms; *p* = 0.61). **(C)** The RRP consisted of 705 ± 92 and 156 ± 18 synaptic vesicles (SVs) in AP and BP with a ratio of 37 aF/SV, respectively (*p* < 0.001). **(D)** There was no significant difference in the number of RRP vesicles triggered by per unit Ca^2+^ influx in AP and BP hair cells (1.79 ± 0.431 SVs/pA vs. 2.18 ± 0.517 SVs/pA; *p* = 0.57). **(E)** SRR from AP hair cells (5,615 ± 800 SVs/s) was remarkably faster than that from BP (1,063 ± 200 SVs/s). (*p* < 0.001). **(F)** Dividing SRR by *I*_Ca_ peak indicated AP has a larger rate of SRP release triggered by per unit Ca^2+^ influx than BP (18.5 ± 3.27 SVs/s⋅pA vs. 11 ± 1.35 SVs/s⋅pA; *p* = 0.04). Data are presented as mean ± SEM (*p < 0.05, **p < 0.01, ***p < 0.001; unpaired Student’s *t*-test).

To examine the dynamics of exocytosis, we varied the length of voltage steps from 2 ms to 1 s and used curve fitting to extract RRP and sustained release rate (SRR, refer to the R_sustained_ in section “Materials and methods”; Figure 5A). Assuming a single vesicle capacitance of 37 aF (Lenzi et al., 1999), we found that the RRP consisted of 705 ± 92 (*n* = 12) and 156 ± 18 (*n* = 14) synaptic vesicles for AP and BP hair cells (Figure 5C), respectively. The time constants (τ) for the depletion of this fast pool were 17.7 ± 4.71 and 14.2 ± 4.92 ms for the responses of AP and BP hair cells (Figure 5B), respectively, which showed no significant difference (*p* = 0.61, unpaired Student’s *t*-test). Subsequently, an estimation of the sustained release rate (SRR) was obtained by fitting the linear range of the data. We found that the average SRR was 5,615 ± 800 SVs/s and 1,063 ± 200 SVs/s in AP and BP hair cells (Figure 5E), respectively, from which it can be learned the release rate of the latter that is about five-folds greater than the former. No significant difference was observed for τ to release RRP while the other two parameters (RRP and SRR) were both significantly reduced in BP hair cells, suggesting that its function of exocytosis was comparatively weak. We then divided the vesicles in the total RRP by Ca^2+^ current, and the results represented the number of RRP vesicles triggered per unit Ca^2+^ influx, showing no significant difference in these two auditory hair cells (1.79 ± 0.431 SVs/pA vs. 2.18 ± 0.517 SVs/pA, *n* = 12 and 14 for AP and BP hair cells; *p* = 0.57; Figure 5D). However, it existed a significant difference in SRR/*I*_Ca_ between AP (18.5 ± 3.27 SVs/s⋅pA) and BP (11 ± 1.35 SVs/s⋅pA) hair cells (unpaired Student’s *t*-test, *p* = 0.04; Figure 5F).

**FIGURE 6:**
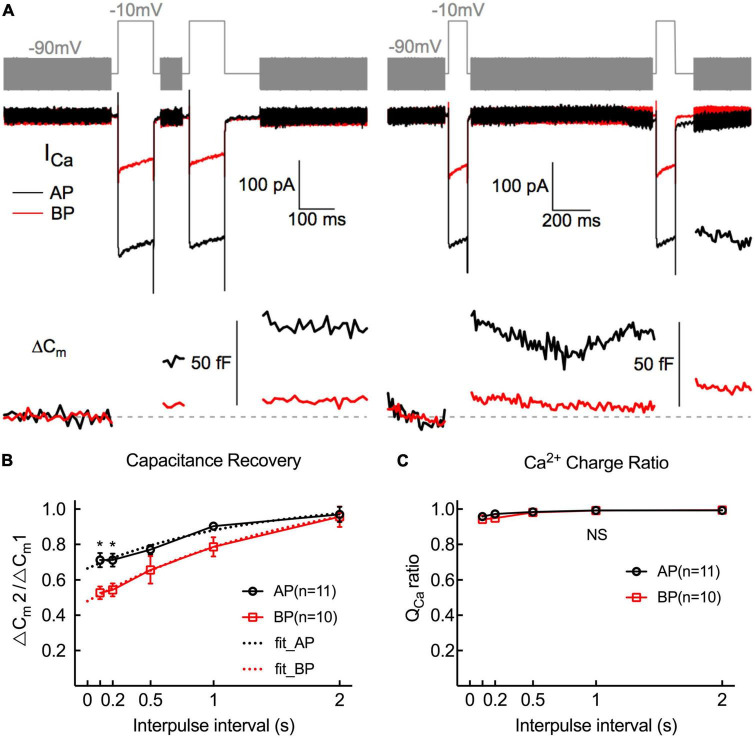
Replenishment of synaptic vesicles from hair cells Paired pulses (depolarized to –10 mV lasting 100 ms) with variable intervals (from 100 ms to 2 s) between both stimuli were used to measure the time course of endocytosis in AP and BP hair cells (*n* = 11 and 10). **(A)** Two paradigms of C_m_ traces and Ca^2+^ current responses with intervals of 100 ms (left) and 1 s (right). The double pulses induced notable *I*_Ca_ and ΔC_m_. **(B)** The fraction of the ΔC_m_ increment (ΔC_m2_/ΔC_m1_) elicited by the first and second depolarizations at certain intervals was calculated to quantify the synaptic vesicle replenishment. Synaptic vesicle replenishment was significantly faster in AP than in BP hair cells at intervals of 100 ms and 200 ms. * means *p* < 0.05. **(C)** The ratio of Ca^2+^ charge in two stimulation durations showed no significant difference in both cell types.

To study the kinetics of vesicle replenishment after voltage-gated Ca^2+^ entry, we measured the recovery of the RRP from the depletion by using paired stimuli (two times 100 ms depolarizations to −10 mV) separated by various intervals (from 100 to 2,000 ms) (Figure 6B). Relatively short stimuli reveal that the RRP and longer steps induce sustained release (Parsons et al., 1994; Schnee et al., 2005). We depolarized hair cells for 100 ms, designing to ensure depleting RRP vesicles. Noting that it showed a larger first than second ΔC_m_, which might be due to Ca^2+^ current inactivation, vesicle depletion, or postsynaptic glutamate receptor desensitization.

The ratio of the capacitance increases (ΔC_m2_/ΔC_m1_) in response to the paired step depolarizations was used to assess the synaptic vesicle replenishment. As shown in Figure 6B, the synaptic vesicle replenishment in BP was significantly slower with an interval as short as 100 and 200 ms (100 ms: AP: 0.712 ± 0.039, BP: 0.527 ± 0.037; 200 ms: AP: 0.712 ± 0.036, BP:0.544 ± 0.037; *n* = 11 and 10; two-way ANOVA followed by Bonferroni *post-hoc* test, *p* < 0.05), and no significant difference was observed between the two groups for the other intervals, despite the average ratio of AP showed a larger tendency. We then fitted the recovery curves with a one-phase association equation and obtained the recovery time constants (AP: tau = 1.247 s, BP: tau = 1.746 s). The complete recovery time for capacitance from paired-pulse depression in BP hair cells was greatly delayed compared to AP [*F*(1,19) = 13.7, *p* = 0.002, two-way ANOVA followed by Bonferroni *post-hoc* test]. Furthermore, we calculated the Ca charge ratio to evaluate the Ca^2+^ influx during the process of paired stimulus and found no significant difference in both cell types [*F*(1,20) = 0.787, *p* = 0.39, two-way ANOVA followed by Bonferroni *post-hoc* test], which excluded the consequence resulted from the difference in Ca^2+^ influx. Given the quick capacitance recovery in AP hair cells, it is probably ascribed to the high rate of vesicle replenishment, and the underlying mechanism needs further investigation.

## Discussion

The remarkable acuity and temporal precision of the auditory system relay to exocytosis at the hair cell’s ribbon synapses, which are specialized to operate at their most suitable frequencies. Analogous to other vertebrate auditory organs, bullfrogs have hair cells arranged in a tonotopic axis. Our study provides a quantitative description of vesicle release from AP and BP hair cells in the adult bullfrog, which are responding to comparatively lower and higher frequencies. We found that the properties of ionic currents and kinetic exocytosis from hair cells in these two auditory organs differed dramatically.

Compared to amphibian species, the turtle’s BP is composed of 900 hair cells (Hackney et al., 1993), which are arranged tonotopically with resonant frequencies varying from approximately 40 to 500 Hz (Crawford and Fettiplace, 1980). The low-frequency hair cells are located toward the apical or lagenar end and high-frequency cells are close to the basal or saccular end. Variations in resonant frequency might be associated with the changes in the number of hair cell transmitter release sites and Ca^2+^ channel density along the tonotopic axis of the cochlea (Sneary, 1988; Martinez-Dunst et al., 1997). It showed an exponential increase in peak Ca^2+^ current with fractional distance along the basilar papilla from the low-frequency end (Ricci et al., 2000). Turtle cochlear hair cells are electrically tuned by a voltage-dependent Ca^2+^ current and a large-conductance Ca^2+^-dependent K^+^ current (BK_(Ca)_) (Art and Fettiplace, 1987; Ricci et al., 2000). Cells are tuned to higher frequencies possessing more channels of Ca^2+^ and K^+^ than low-frequency cells (Wu et al., 1995). Furthermore, outward K^+^ current in low-frequency cells (<30 Hz) shows less sensitivity to TEA, which is pharmacologically distinct from that in high-frequency cells (Goodman and Art, 1996).

Considering fast and slow voltage-dependent potassium currents are selectively, spatially distributed within the epithelium in amphibians, reptiles, and birds (Smotherman and Narins, 1999b), the different potassium current types may underlie the observed variance. Specifically, in both types of hair cells, aside from the calcium-dependent outward potassium current *I*_K(Ca)_, which accounts for the majority of the component of outward ionic current, the slowly activating *I*_K_ is also the predominant outward current in medial AP hair cells, while the component of fast-inactivating *I*_A_ is a feature of BP hair cells, which might make for a better function corresponding to the resonances and handle higher frequency auditory information. The role of *I*_A_ in hearing formation is probably related to its rapid inactivation, and the regulation of cell excitability through transient hyperpolarization membrane potential, which implies potassium channel, might play an essential role in the auditory encoding process.

According to the previous study, from a holding potential of −60 mV, *I*_K(Ca)_ dominates the outward current and most of *I*_K_ is inactive (Smotherman and Narins, 1999b), and with increasing depolarization, *I*_K_ will slowly overcome its inactivation during prolonged depolarizations, adding a slowly activating component to the net outward current, which can explain the minority proportion of *I*_K_ in our study. In turtles, *I*_K_ in hair cells tuned to low frequencies was carried by a single class of channels with an apparent affinity for TEA. When tuned to higher frequencies, *I*_K_ was carried by a single type of potassium channel with a high affinity for TEA and a low affinity for 4-AP (Goodman and Art, 1996). Consistently, we found that in the bullfrog’s hearing organ, *I*_K_ in the medial AP hair cells that tune to lower frequencies showed a higher sensitivity to TEA. What is more, the outward components in medial AP hair cells exposed to 10 mM TEA were greatly reduced, supporting the outward current carrier likely to be the *I*_K_. Furthermore, the different cell sizes may contribute substantially to the observed variance in Ca current and exocytosis. As has been noted, the *I*_Ca_ peak in response to depolarization and the ensuing increased ΔC_m_ is greater in AP hair cells when compared to BP, probably due to the fewer synaptic active zones in BP synapses. The possible reasons for the smaller *I*_Ca_ amplitude in BP hair cells could be fewer numbers of Ca^2+^ channels expressed on the cell membrane. We then found that Ca^2+^ channels in AP show a hyperpolarizing tendency and stronger voltage dependence in Ca^2+^ current activation, associated with a significantly negative V_half_ and a steeper *k*, matching well with the negative receptor potentials in lower frequency hair cells and large AC component in voltage responses (Russell and Kössl, 1992).

The anuran auditory papillae share some apparent characteristics with the mammalian cochlea (Moser and Beutner, 2000), and at least two kinetically distinct components of vesicle release were evident in adult bullfrog hair cells: a rapidly small RRP and a slower but larger SRP. Notably, vesicles tethered to the ribbon provide a pool for sustained release that is typically five-folds greater than the docked pool available for fast release (Sterling and Matthews, 2005), which is roughly consistent with our results mentioned above. The higher numbers of vesicles released at AP hair cell synapses are indicative of increased temporal precision of exocytosis in lower frequency hair cells of bullfrogs. While in mammalian cochlea such as gerbils, high-frequency cells are more indefatigable than low-frequency cells by virtue of the pronounced tonotopic differences in the Ca^2+^-dependent exocytosis and vesicle pool replenishment at IHC ribbon synapses (Johnson et al., 2008). An unexpected finding in our study was a considerable reduction of ΔC_m_ in BP hair cells, simultaneously accompanied by a remarkably lower RRP. Since the total number of release sites equals the number of vesicles available for the RRP in a hair cell (Graydon et al., 2011), we can surmise the release sites at ribbons or the ribbon number in BP hair cells might be fewer than those in AP. This is contrary to the earlier report (Johnson et al., 2008) in gerbils that the overall number of vesicles from RRP is greater in high-frequency cells though the RRP at individual synapses is similar between both low- and high-frequency cells. We also discovered the high- and low-frequency cells have the same Ca^2+^ efficiency of exocytosis, hence, low-frequency hair cells in bullfrog undertake a greater Ca^2+^ load per synapse, and they also seem to release at a faster rate than high-frequency ones (Patel, 2013). RRP depletion is discussed as a mechanism for fast auditory adaptation (Moser and Beutner, 2000). The RRP of BP hair cells exhibited an averaged RRP depletion time constant of about 14 ms that was comparable to AP hair cells (18 ms), indicating that the release probability of the remaining readily releasable vesicles was similar in both low- and high-frequency hair cells. The total number of vesicles from RRP was smaller in BP cells, yet τ to release RRP was not significantly differed at all, suggesting that high-frequency hair cells deplete the rapidly releasable vesicles faster than that low-frequency hair cells in the bullfrog. Possible explanations for this could be a lower cytoplasmic vesicle density in BP hair cells and/or the poor replenishment of ribbon-associated synaptic vesicles.

Ribbon synapses at bullfrog figure prominently in the sustained release of hundreds of synaptic vesicles as well. Thus, fast and efficient vesicle replenishment is highly necessary to maintain a high rate of exocytosis lastingly (Pangršič et al., 2010). As shown in Figure 6A, a pair of 100 ms, equally strong depolarizations, evoked the paired-pulse depression. It is worth noting that the two Ca^2+^ currents are virtually identical (Figure 6C), demonstrating that secretory depression occurred (Moser and Beutner, 2000), though Ca^2+^ inflowed without attenuation. All the low (AP) and high (BP) frequencies, hair cell synapses showed a paired-pulse depression at relatively shorter intervals and then gradually recovered, suggestive of variations in the rates or ability of endocytic membrane retrieval and the feeble function of synaptic vesicle refilling in high-frequency hair cell (BP) synapses. The comparatively larger RRP release and higher sustained release rate (SRR) of synaptic ribbons at AP reveal its increased temporal precision of exocytosis and faster replenishment of synaptic vesicles, which may make for the rapid capacitance recovery.

To summarize, we found a biophysical trend that distinguishes BP hair cells from AP in this study. Commonly, BP hair cells have small steady-state outward potassium currents that inactivated rapidly. The fact is that the currents from BP inactivate faster than AP, which suits well to the different frequencies of sound that they are responding to. Moreover, we conclude the notable tonotopic differences in the kinetics and Ca^2+^ sensitivity of exocytosis from bullfrog hair cells. The synaptic machinery seems to be especially designed for sustaining neurotransmitter release with lower frequency cells being more steady and sufficient.

## Data availability statement

The original contributions presented in this study are included in the article/supplementary material, further inquiries can be directed to the corresponding author.

## Ethics statement

The animal study was reviewed and approved by the Shanghai Medical Experimental Animal Administrative Committee.

## Author contributions

JZ and NY performed the experiments and analyzed the data. JZ drafted the manuscript. G-LL acquired the funding, provided guidance in experimental designs, helped in experimental troubleshooting, and edited the manuscript. All authors contributed to the article and approved the submitted version.
